# WHO’S SARCOPENIC? AN ANALYSIS USING THE CANADIAN LONGITUDINAL STUDY ON AGING DATA

**DOI:** 10.1093/geroni/igae098.1957

**Published:** 2024-12-31

**Authors:** Stuart Phillips

**Affiliations:** McMaster University, Hamilton, Ontario, Canada

## Abstract

A limitation of the sarcopenia definitions used in our analyses is the use of DXA measured ALM to approximate muscle mass. Although many consider ALM to be the reference standard for measuring muscle mass for sarcopenia, it is actually a measure of lean mass that includes organ tissue, water, and all other non-bone and non-fat soft tissues in addition to muscle mass. The results may be substantially altered if more accurate measures of muscle mass such as the D3-creatine method were used. We have reported that there is generally slight to moderate agreement (Cohen’s κ values of.00–.60) between most of the combinations of variables used to ascertain sarcopenia status recommended by the expert group definitions. For definitions using lean mass, the agreement between different adjustment techniques for lean mass ranged from slight to substantial. These findings were consistent across the range of cutoffs for each variable that are either observed in the literature or recommended by the expert group definitions for sarcopenia. The general lack of agreement between sarcopenia definitions observed in our study underscores the importance of the sarcopenia research community identifying a unified ascertainment method for sarcopenia. Having multiple definitions of sarcopenia identifying different groups of individuals as sarcopenic may hinder vital research developing clinical management strategies for sarcopenia by decreasing the comparability between studies.

